# Mediation model of comorbid anxiety disorders in late-life depression

**DOI:** 10.1186/s12991-020-00313-3

**Published:** 2020-11-17

**Authors:** Chawisa Suradom, Nahathai Wongpakaran, Tinakon Wongpakaran, Peerasak Lerttrakarnnon, Surin Jiraniramai, Unchulee Taemeeyapradit, Surang Lertkachatarn, Suwanna Arunpongpaisal, Pimolpun Kuntawong

**Affiliations:** 1 Geriatric Psychiatry Unit, Department of Psychiatry, Faculty of Medicine, Chiang Mai University, 110 Intawaroros Rd., Chiang Mai, 50200 Thailand; 2 Department of Family Medicine, Faculty of Medicine, Chiang Mai University, Chiang Mai, Thailand; 3 Songkhla Rajanagarindra Psychiatric Hospital, Songkla, Thailand; 4 Prasat Neurological Institute, Bangkok, Thailand; 5 Department of Psychiatry, Faculty of Medicine, Khon Kaen University, Khon Kaen, Thailand

**Keywords:** Anxiety disorder, Late-life depression, Neuroticism, Mediation

## Abstract

**Background:**

A number of studies have been conducted on risk factors of comorbid anxiety disorders regarding late-life depression (LLD). This study investigated the associated factors and their relationship to comorbid anxiety disorders in LLD.

**Methods:**

Participants included 190 elderly Thais (73.2% female, with a mean age of 68.39 ± 6.74 years) with depressive disorders, diagnosed according to DSM-IV Diagnosis Axis I disorders assessed by Mini-International Neuropsychiatric Interview. Demographic data, medical and psychiatric history, family psychiatric history, past depression, family history of depression, Neuroticism Inventory and 7-Item Hamilton Depression Rating Scale (HAMD-7) were analyzed for path analysis using Structural Equation Model framework. The bootstrapping method was used for testing indirect effects.

**Results:**

Being female was associated with comorbid anxiety disorders with an indirect effect (*β *= − 0.032, *P* = 0.018) through neuroticism, depression severity, history and family history of depression. Family history of depression had no effect on comorbidity (*P* = 0.090). Neuroticism had an indirect effect on comorbid anxiety disorders (*β* = 0.075, *P* = 0.019) via depression severity as reflected by HAMD-7 score (*β *= 0.412, *P* =  < 0.001). Total variance explained from this model was 11%. This model had good-fit index with Chi-square > 0.05, CFI and TLI > 0.95 and RMSEA < 0.06.

**Conclusion:**

Neuroticism mediates the effect of relationship between sex, family history and history of depressive disorders and comorbid anxiety disorders in LLD. Moreover, depression severity is a mediator for neuroticism and comorbid anxiety disorders. Longitudinal studies are warranted to indicate the importance of effective treatment of depression to lower the risk of developing comorbid anxiety disorders among depressed elderly.

## Background

Anxiety is common in late-life depression (LLD), both as a symptom and as a comorbid disorder [1]. Comorbid anxiety disorders have been found to increase the burden of depression as reflected by quality of life, physical disability, increased health care use and mortality [2]. According to related studies, comorbid anxiety disorder was associated with poorer social function, a higher level of somatic symptoms, a higher level of suicidality [1], a higher risk for alcohol abuse [3], disability [4] and lower cognitive performance [5].

A number of studies have been conducted on risk factors of comorbid anxiety disorders regarding LLD. Evidence has proved a significant correlation of history of depressive disorders and comorbid anxiety and depression. Specifically, past depressive disorders increased the odds of repeated anxiety disorders, depressive disorders and of repeated comorbid anxiety and depressive disorders [6].

Apart from history of depressive disorder, the associated factors of the comorbidity identified by related studies included, being female [7], higher level of depression severity [8] and personality trait neuroticism [9], while results for family histories of mental disorder as a risk were inconclusive [10], despite the fact that several studies have identified genetic correlation to the emergence of depressive and anxiety disorders [11–13]. None however, has investigated the role of family history in the comorbidity of anxiety and depressive disorders.

Notably, personality trait neuroticism is a predisposing variable to developing many psychiatric disorders, especially anxiety and depressive disorders. One particular study attempted to impose a mediation model on the theory with findings that included neuroticism substantially for a number of anxiety disorders, namely, GAD, panic disorder and phobias [9]. The role of neuroticism on depression was more clearly demonstrated in elderly populations [14, 15].

Based on these aforementioned associated variables, evidence showed that comorbid anxiety disorders were influenced by sociodemographic factors such as sex, biologically predisposing factors such as history of depression and family history of depression and psychological factors such as personality trait neuroticism. What is lacking is to include all these variables addressing bio-psychosocial issues and test them simultaneously.

**Fig. 1:**
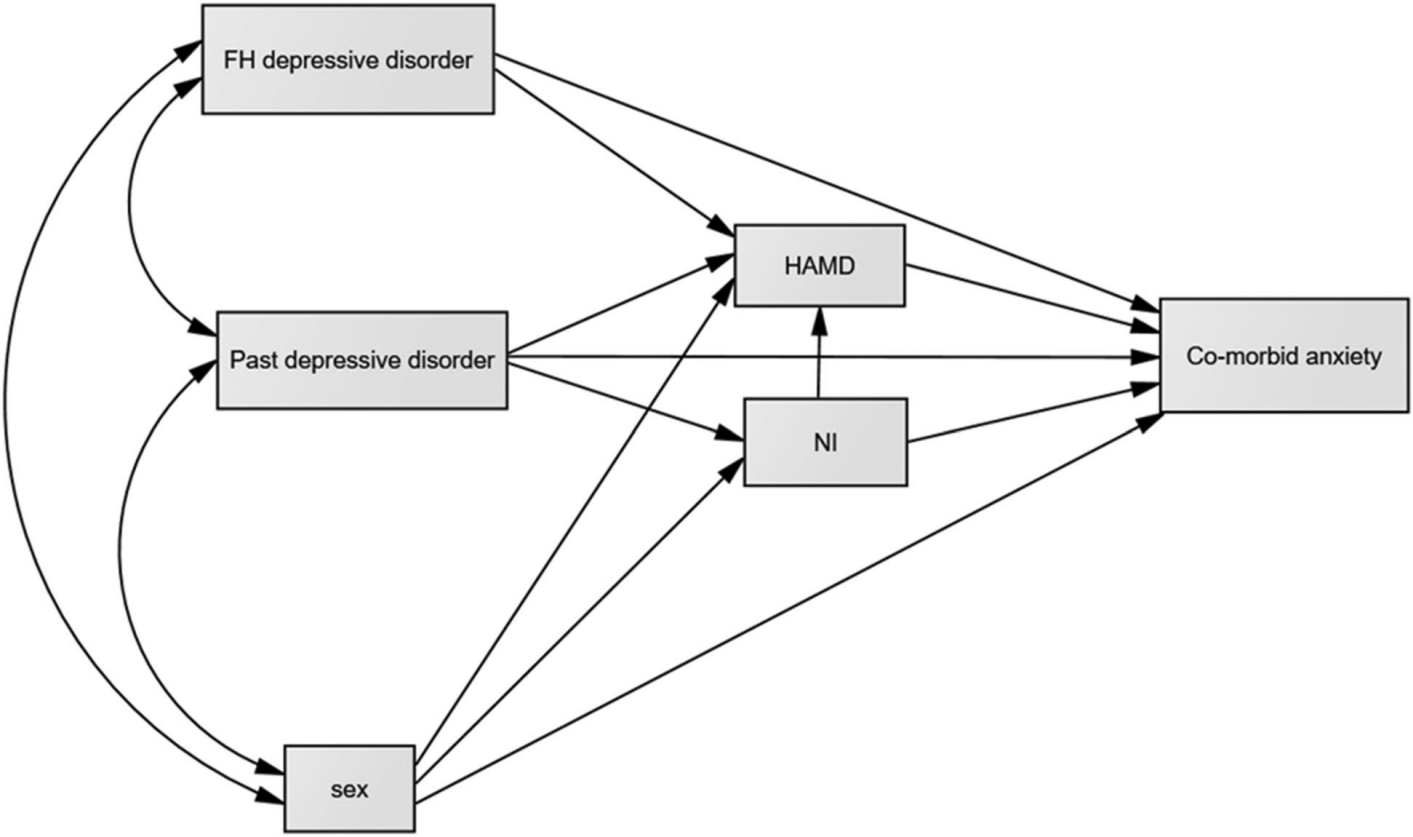
Hypothesized model of comorbid anxiety and depressive disorders. *NI* neuroticism Inventory, *HAMD* Hamilton Depression Rating Scale, *FH* family history of depressive disorder. The lines with arrowheads show the direction of the path coefficients

We hypothesized that these independent variables (individual’s history of depression, family history of depression and being female) would have a positively direct effect on comorbid anxiety disorders, regarded as dependent variables. We hypothesized that neuroticism and level of depression severity should serve as mediators of the relationship between independent and dependent variables; thereby, three independent variables, two mediators and a single outcome rendered eight mediational models (Fig. 1). However, as recommended by Hayes, testing all variables as a single integrated model is more worthwhile than testing each separately because it would be interesting to find the strength of association of each paired variable [16]. From the single model, we hypothesized that the level of neuroticism and depression severity would mediate the direct effect of past depression, family history of depression or sex on comorbid anxiety disorders.

Moreover, none of the related studies on the associated factors of comorbid anxiety disorders has been conducted regarding LLD. Therefore, this study aimed to investigate the associated factors and their relationship on comorbid anxiety disorders concerning LLD.

## Methods

This research comprised a secondary analysis of data of elderly Thai patients with a diagnosis of depressive disorders between October 2012 and March 2015 [17].

### Participants

Psychiatric elderly patients were evaluated at four different tertiary care centers across Thailand, namely, Maharaj Nakorn Chiang Mai Hospital, Prince of Songkla University Hospital, Songkla Rajanagarindra Psychiatric Hospital and Prasat Neurological Institute using the Mini-International Neuropsychiatric Interview (MINI) to evaluate major Axis I diagnoses according to DSM-IV or ICD-10 criteria [17]. A total of 190 patients with depressive disorders were then evaluated for comorbid anxiety disorders and their associated factors.

Information regarding demographic characteristics and symptoms were gathered and evaluated to identify risk factors for comorbid anxiety disorders in LLD. Inclusion criteria included new cases of patients aged 60 and beyond who attended the psychiatric outpatient unit with one of the following symptoms: sadness, loss of interest, sleep disturbance, poor appetite, memory problems, lack of energy and unexplained medical symptoms. The exclusion criteria included those with medical conditions that interfered with the interview process, illiteracy or language barrier, cognitive impairment, history of schizophrenia, schizo-affective or mania with residual symptoms [17].

LLD is defined as depressive disorders among patients aged 60 and above, receiving treatment in tertiary care centers, as diagnosed by the Mini-International Neuropsychiatric Interview (MINI) for major depressive disorders (MDD) based on DSM-IV TR. Anxiety disorders consist of panic disorders, agoraphobia, social phobias and generalized anxiety disorders. Other related disorders included obsessive–compulsive disorders and post-traumatic stress disorders.

### Measurements

Demographic data, medical and psychiatric history, family psychiatric history and a number of measurements included those listed below.

#### Mini-International Neuropsychiatric Interview (MINI)

Mini-International Neuropsychiatric Interview (M.I.N.I., 5.0.0) is a semi-structured interview for diagnosing psychiatric disorders based on the DSM-IV. Depressive disorders, i.e., major depressive disorders (MDD) and dysthymia, were in Modules A and B. The Thai version has been validated and widely used [18].

#### The 7-item Hamilton Depression Rating Scale (HAMD-7) [19].

The HAMD-7 is a semi-structured interview for diagnosing severity of depression based on the DSM-IV. We employed the GRID version of HAMD-7, for which the researchers were trained until the inter-rater agreement was ensured to reach a 100% agreement on the scoring.

#### Neuroticism Inventory (NI) [20]

The NI is a self-reporting assessment tool for measuring dimensionality of the neuroticism personality trait according to Eysenck’s Five-factor Model [21]. The NI, developed by Wongpakaran et al., includes 15 items with a 0 to 4 Likert scale [20]. A higher score reflects a higher level of neuroticism. It has been demonstrated as a valid and reliable tool. The present study yielded a Cronbach’s alpha of 0.83.

### Statistical analysis

Descriptive statistics were used to assess demographic data such as sex and age. Correlations among variables were analyzed using Pearson’s, point-biserial, and Spearman’s rank correlations. All demographic data, medical and psychiatric history, and measurement scores were analyzed to identify meaningful correlations. The main outcome in this study was comorbid anxiety-depressive disorder. Sociodemographic data served independent variables. Mediator variables included neuroticism and severity of depressive symptom, and significant variables were included in the model. Path analysis in the structural equation model framework was used to build the interrelationships among all variables.

Following steps were carried out. The first step involved exploring the relationship between independent and dependent variables using Pearson’s and point-biserial correlation to ensure significant relationship among variables to be candidate predictors and mediators for the comorbid anxiety-depressive disorder. Then we examined the relationship among all potential predictors using path analysis. The hypothetical relationships were evaluated to test how neuroticism and severity of depression may mediate the pathway from sociodemographic variables to comorbid anxiety-depressive disorder. Model fit was evaluated using the following criteria: Chi-square/*df* < 3, Comparative Fit Index > 0.95, Tucker–Lewis Index > 0.95 and root mean square error of approximation (RMSEA) < 0.06. In model comparison, Consistent Akaike information criterion (CAIC) was used to compare two or more not-nested models, with smaller values representing a better fit, and Bayesian Information Criteria (BIC) was also used to determine the best-fit model as it has a greater tendency to pick parsimonious models. Finally, we evaluated whether the putative mediators, according to the best-fit model, could significantly mediate the effect using the bias-corrected and accelerated (BCa) bootstrapped method to calculate the empirical significance level (based on 10,000 bootstrap replications).

The data were analyzed using IBM SPSS for Windows, Version 22.0 (IBM Company, Armonk, NY, USA); AMOS, Version 22 was used for the path model.

## Results

Demographic data and history of depression are shown in Table 1. The overall prevalence of anxiety disorders, also diagnosed with MINI, in this elderly population with LLD was 16.84%, with GAD and agoraphobia being the most prevalent. Significant correlated variables from literature review, as identified by Pearson’s correlation, were sex, history and family history of depressive disorder, Neuroticism Inventory score, measuring neuroticism personality trait and HAMD-7 scores, measuring depression severity.

**Table 1:** Demographic data and current psychiatric history

Demographic data	*N* (%)
Female gender	139 (73.2)
Age (years), mean (SD); min–max	68.39 (6.7); 60–88
Marital living status	
Living alone	75 (39.5)
Living with spouses	115 (60.5)
Years of education, mean (SD); min–max	6.67 (5.0); 0–30
History of depressive disorders	27 (14.2)
Family history of depressive disorders	7 (3.7)
Concurrent medical conditions	52 (27.4)
Anxiety disorders	32 (16.8)
Panic disorder	9 (4.7)
Agoraphobia	10 (5.3)
Social phobia	2 (1.1)
OCD	4 (2.1)
PTSD	7 (3.7)
GAD	14 (7.4)
Measurements, mean (SD)	
MoCA	15.70 (5.473)
HAMD-7	10.30 (6.340)
NI	53.44 (11.699)

*OCD* obsessive compulsive disorder, *PTSD* post-traumatic stress disorder, *GAD* generalized anxiety disorder, *MoCA* Montreal Cognitive Assessment, *NI* neuroticism inventory, *HAMD-7* 7-Item Hamilton Depression Rating Scale

According to Baron, correlation between independent (*X*) and outcome (*Y*), and *X* and *M* has to be observed [22]. Table 2 shows significant correlations for being female and depressive history, neuroticism, comorbid anxiety disorders, and depression severity. Depressive disorder also correlated with neuroticism, comorbid anxiety disorders and depression severity. Interestingly, comorbid anxiety disorders significantly correlated with family history of depressive disorder. In contrast, neuroticism and depression severity correlated with all variables, except for family history of depressive disorder.

**Table 2:** Zero-order correlations between variables

Variable	Gender	Past depressive disorders	Family history of depressive disorders	Neuroticism	Comorbid anxiety	HAMD-7
Gender	1					
Past depressive disorders	− 0.173	1				
Family history of depressive disorders	− 0.055	0.100	1			
Neuroticism	− 0.214**	0.159*	0.049	1		
Comorbid anxiety	− 0.146*	0.143*	0.211**	0.189**	1	
HAMD-7	− 0.181*	0.313**	0.088	0.462**	0.234**	1

^*^p < .05, **p < .01, ***p < .001; *GDS* Geriatric Depression Scale, *HAMD-7* the 7-Item Hamilton Depression Rating Scale, *MoCA* Montreal Cognitive Assessment

We hypothesized that depression severity, family and history of depressive disorder, being female and neuroticism all had an effect on comorbid anxiety disorder in LLD, while the latter four also exhibited an effect through depression severity as a mediator. Moreover, we suspected the mediation effect of neuroticism on sex and history of depressive disorder.

### Mediation analysis

**Fig. 2:**
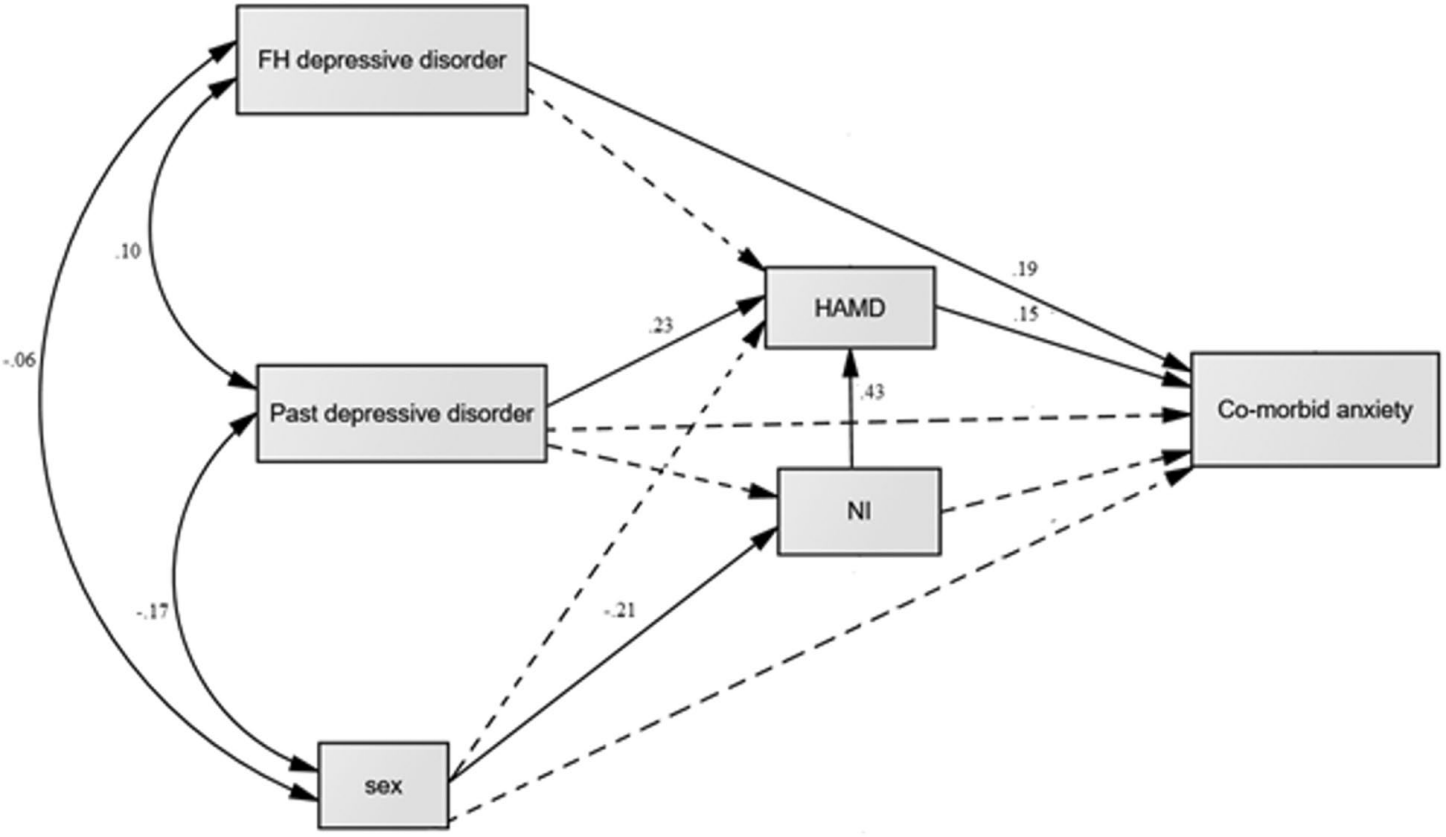
The final path model illustrating direct and indirect effects and causal paths linking variables with comorbid anxiety and depressive disorders. *NI* neuroticism Inventory, *HAMD* Hamilton Depression Rating Scale, *FH* family history of depressive disorder. The lines with arrowheads show the direction of the path coefficients. Values on the lines = significant path coefficient or standardized coefficient. Dash lines denotes nonsignificant path coefficient or standardized coefficient and the actual values omitted

The proposed mediation model satisfied a good-fit model to the data with the *χ*^2^ (*df*) = 0.288, RMSEA = 0.000, TLI = 1.122, CFI = 1.000, CAIC = 119.270, BIC = 100.270. However, to find the best model, CAIC was used to compare other models in which nonsignificant paths were deleted one by one. The final model yielded the best fit with the lowest CAIC while showing other fit indices above the cut-off criteria (*χ*^2^ (*df*) = 1.136, RMSEA = 0.027, TLI = 0.977, CFI = 0.989, CAIC = 95.411, BIC = 81.411) (Fig. 2).

### Testing for mediation model

From Table 3, only HAMD showed a direct effect on comorbidity of anxiety disorders in LLD (*β* = 0.215, BCa 95% CI = 0.107–0.316). Neuroticism Inventory score, history of depression, and being female predicted the comorbidity of anxiety disorders in LLD with indirect effects of *β* = − 0.053, *P* = 0.018, *β* = 0.092 (BCa 95% CI = 0.041–0.153), *β* = − 0.020 (BCa 95% CI = − 0.046 to − 0.007), respectively. Family history of depressive disorder itself had a nearly significant effect on the comorbidity (*P* = 0.061).

**Table 3:** The standardized coefficients and bias-corrected 95% confidence interval of effect of variables on mixed anxiety-depressive disorder

Variables	Total effect	*p*-value	Direct effect	*p*-value	Indirect effect	*p*-value
Gender	− 0.020 (− 0.046, − 0.007)	0.002	–	–	− 0.020 (− 0.046, − 0.007)	0.002
Past history of depression	0.053 (0.025, 0.097)	0.001	-	–	0.053 (0.025, − 0.094)	0.001
Family history of depression	0.192 (0.021, 0.354)	0.060	0.192 (0.021, 0.354)	0.060	–	–
HAMD score	0.215 (0.107, 0.316)	0.001	0.215 (0.107, 0.316)	0.001	–	–
Neuroticism	0.092 (0.041, 0.153)	0.001	–	–	0.092 (0.041, 0.153)	0.001

## Discussion

The present study confirmed our hypothesis of various variables associated with the comorbidity of anxiety disorder in LLD. Firstly, depression severity plays a role on the comorbidity of anxiety disorder. Several studies have focused on the effect of anxiety on depression severity with established knowledge in terms of neuro-endocrinology, especially cortisol increasing the risk of depression. No study to date, however, has mentioned the reversed role of depression severity affecting the emergence of comorbid anxiety disorders. Suspected explanations for this might be the same effect of neurobiologic change of the HPA axis or neurotransmitter, together with common psychosocial factors that contribute to both depression and anxiety.

Secondly, neuroticism mediates various variables, namely sex, depressive history and family history of depressive disorder. With neuroticism as a “trait” that persists throughout one’s life, it would be inevitable that this personality trait plays many roles across different aspects of a person’s psychological making. Being female has been found to be associated with higher level of neuroticism, both in younger and older age groups [23], which could potentially explain the higher prevalence of comorbid anxiety disorder among older women compared with men. How neuroticism is associated with anxiety has been studied to an extent, among Western and Asian populations confirming its role in this contribution. Evidence suggests that neuroticism predisposes to both anxiety and depression. One interesting theory that endeavored to explain this association was how genetic factors overlapped between anxiety and neuroticism, resulting from a twin study applying the structural equation model [13]. With this theory in mind, it would be likely that genetics might also play a role in the mediation effect of family history of depressive disorder. Similarly, with neuroticism likely to persist through one’s life span, and evidence shown that it is a strong risk factor for depression [24], neuroticism contributes to history of depressive disorder playing a role in the relationship with comorbid anxiety disorder.

Lastly, family history of depressive disorder had a direct effect on the comorbidity, possibly due to genetic susceptibility of both psychiatric disorders recently believed to be on the same spectrum. Molecular genetic studies have identified specific genetic loci that may have influenced this susceptibility, albeit in their preliminary stages [13].

This study is among the few and most extensive studies on the comorbidity of anxiety disorders in LLD among Asians. The strength of this study includes the fact that we used data from a respectable study conducted in tertiary care centers, including as many bio-psycho-social factors as were identified in related literature. Moreover, we created a path model to investigate further relationships among variables and tested our hypothesis regarding the mediation model. The main limitations of this study revolved around the cross-sectional design that limited a claim of causal relationship, and longitudinal study that should be conducted next. In addition, some of the data that could potentially have been included to explain our hypothesis were lacking, for example, family history of anxiety disorder. Further studies with more extensive data would prove beneficial.

## Conclusion

Neuroticism is an important mediator of comorbid anxiety disorder in LLD with effects on sex, family history and history of depressive disorder, providing supportive evidence for a theory emphasizing the complex relationships among depression, anxiety and neuroticism. This finding shows that neuroticism, strongly linked to anxiety, fear, worry and frustration, core components of all anxiety disorders, as well as depressed mood, explains why some variables are related to the increased rate of comorbidity. Moreover, depression severity is a mediator for neuroticism, as reflected by HAMD-7 scores. A longitudinal study should be conducted to further ascertain the effect of these variables that could be used to create appropriate interventions to lower the risk of developing comorbid anxiety disorders.

## Data Availability

The datasets used and/or analyzed during the current study are available from the corresponding author upon reasonable request as data sharing is subject to Ethics Office approval.

## Abbreviations

M.I.N.I.: Mini-International Neuropsychiatric Interview
NI: Neuroticism inventory
HAMD-7: The 7-item Hamilton Rating Scale for Depression
SEM: Structural equation modeling
CFI: Comparative fit index
TLI: Tucker–Lewis index
RMSEA: Root mean square error of approximation
CAIC: Consistent Akaike information criterion (CAIC)
BIC: Bayesian information criteria
BCa: The bias-corrected and accelerated
